# Decreased Thalamocortical Connectivity in Chronic Ketamine Users

**DOI:** 10.1371/journal.pone.0167381

**Published:** 2016-12-15

**Authors:** Yanhui Liao, Jinsong Tang, Jianbin Liu, An Xie, Mei Yang, Maritza Johnson, Xuyi Wang, Qijian Deng, Hongxian Chen, Xiaojun Xiang, Tieqiao Liu, Xiaogang Chen, Ming Song, Wei Hao

**Affiliations:** 1 Institute of Mental Health of the Second Xiangya Hospital of Central South University, The China National Clinical Research Center for Mental Health Disorders, National Technology Institute of Psychiatry, Key Laboratory of Psychiatry and Mental Health of Hunan Province, Changsha, Hunan, China; 2 Department of Psychiatry and Biobehavioral Sciences, UCLA, 760 Westwood Plaza, Los Angeles, United States of America; 3 Department of Radiology, The People's Hospital of Hunan Province, Changsha, China; 4 Department of Addiction Medicine, Hunan Brain Hospital, Changsha, China; 5 Department of Addiction Medicine, Shenzhen Mental Health Center, Shenzhen Kangning Hospital, Shenzhen, China; 6 Brainnetome Center and National Laboratory of Pattern Recognition, Institute of Automation, Chinese Academy of Sciences Beijing, China; University of Cambridge, UNITED KINGDOM

## Abstract

Disintegration in thalamocortical integration suggests its role in the mechanistic ‘switch’ from recreational to dysregulated drug seeking/addiction. In this study, we aimed to address whether thalamic nuclear groups show altered functional connectivity within the cerebral cortex in chronic ketamine users. One hundred and thirty subjects (41 ketamine users and 89 control subjects) underwent rsfMRI (resting-state functional Magnetic Resonance Imaging). Based on partial correlation functional connectivity analysis we partitioned the thalamus into six nuclear groups that correspond well with human histology. Then, in the area of each nuclear group, the functional connectivity differences between the chronic ketamine user group and normal control group were investigated. We found that the ketamine user group showed significantly less connectivity between the thalamic nuclear groups and the cortical regions-of-interest, including the prefrontal cortex, the motor cortex /supplementary motor area, and the posterior parietal cortex. However, no increased thalamic connectivity was observed for these regions as compared with controls. This study provides the first evidence of abnormal thalamocortical connectivity of resting state brain activity in chronic ketamine users. Further understanding of pathophysiological mechanisms of the thalamus in addiction (ketamine addiction) may facilitate the evaluation of much-needed novel pharmacological agents for improved therapy of this complex disease.

## Introduction

Ketamine is a “dissociative” anesthetic drug and a derivative of PCP (phencyclidine), but with a shorter duration of action and less toxicity. Ketamine was first synthesized in 1962 by an American chemist Calvin Lee Stevens in the Parke-Davis Laboratories [1, 2]. In 1970 the U.S. Food and Drug Administration (FDA) approved ketamine for human medical use as an anesthetic drug [2]. However, it is also a psychedelic drug that can produce psychological dependence [3]. Recently, ketamine has been used as an illicit drug inside and outside of club and rave scenes in many parts of the world, such as China, Indonesia, Malaysia, and Australia. There are multiple dangers associated with nonmedical use and abuse of ketamine, such as dependence and psychiatric morbidity [4], lower urinary tract dysfunction [5], brain structural [6, 7] and functional [8] abnormality, and sexual impulsiveness or violence [9].

Drug addiction (including ketamine [10–13]) results in deficits in cognitive and emotional processes, which are involved in the thalamocortical network [14]. The thalamus plays a central and dynamic role in information transmission and processing in the brain by controlling corticocortical information that is being passed through the thalamus from one cortical area to another [15, 16]. The thalamus is composed of a very complex circuitry between multiple brain regions, including the prefrontal cortex, anterior cingulate, basal ganglia, cerebellum, motor and sensory regions, and association regions of the cerebral cortex. Thus, the thalamus may play a role as an active partner in the whole brain of cortical computations [16]. Disintegration in the thalamocortical network is a change associated with addiction, it also suggests it plays a mechanistic role in the 'switch' from recreational to dysregulated drug seeking [14].

Resting state functional connectivity is a relatively novel fMRI approach that provides insight for investigations in intrinsic brain connections, critical neurocircuits and pathophysiological alterations in a variety of neuropsychiatric disorders, including schizophrenia and addiction. A functional connectivity study demonstrated the relationship of resting brain hyperconnectivity and schizophrenia-like symptoms produced by ketamine infusion in 22 healthy humans [17]. Understanding chronic use of ketamine and its impact on resting state functional connectivity may help us to understand the neurobiological mechanism for addiction, and given its special role in neuropsychiatric disorders may provide some implications in treatment-resistant depression and schizophrenia [18, 19].

In this study we used rsfMRI to test whether thalamocortical connectivity is altered in chronic ketamine users. Evidence shows that learning and performance factors are integrated into a network centered on the mediodorsal thalamus. Additionally, deficits in goal-directed control and a consequent dysregulation of habit learning processes could result in compulsive drug seeking [14]. Given this evidence we hypothesize that thalamic nuclear groups would show altered resting state functional connectivity (RSFC) in the cerebral cortex of chronic ketamine users (ketamine addicts) when compared with healthy controls.

## Methods

### Study population

One hundred and thirty subjects (41 ketamine dependent subjects and 89 drug-free healthy subjects) were enrolled in this study. All subjects were Han Chinese and between the ages of 19–39 with normal or corrected-to-normal vision. Ketamine dependent volunteers were recruited from two sites in Changsha city: the Kangda Voluntary Drug Rehabilitation Centers in Hunan Province and the Department of Addiction Medicine, Hunan Brain Hospital. All ketamine users met the Diagnostic and Statistical Manual of Mental Disorders (DSM-IV) criteria for lifetime ketamine dependence determined by the Structured Clinical Interview (SCID) [20]. Ketamine subjects were excluded if they met criteria for other substance dependence (excluding nicotine dependence) at any time, past and present. Drug free healthy control subjects were recruited through a combination of targeted site sampling, advertisement, and snowball sampling referrals. All participants were Han Chinese with no history of neurological disorder or other psychiatric illness; neither did they have a first degree relative with substance abuse or a history of psychiatric illness. Additionally, any participant that tested positive for pregnancy was excluded from participation. A licensed psychiatrist conducted all clinical interviews.

The protocol was approved by the university ethics committee (The Second Xiangya Hospital of Central South University Review Board, No. S054, 2008) as well as carried out in accordance with the Declaration of Helsinki. All subjects were informed about all procedures and any potential risks associated with the procedures. Once provided this information, written informed consent was obtained by all subjects. Craving for both ketamine and smoking were assessed by The Visual Analogue Scale for Craving (VASc) [21]. The VASc assesses craving using a scale from 0–10, where a score of 0 represents null craving and a score of 10 represents the most extreme craving. The Digit Symbol Test (DST), Trail Making Test A (TMTA) and The Stroop Color—Word Test (CWT) were also used to measure subject’s cognitive functioning.

### Scan acquisition

MR imaging was carried out using a Siemens Magnetom Trio 3.0T MR scanner (Erlangen, Germany). Functional images were collected axially by using an echo-planar imaging (EPI) sequence sensitive to BOLD contrast. The acquisition parameters were as follows: 36 slices, 3000/30 ms (TR/TE), 4 mm thickness, 220×220 mm (FOV), 64×64 (resolution within slice), 90° (flip angle). The FOV covered all brain regions for all participants. Resting state was collected during the fMRI and lasted 9 minutes with 180 volumes obtained. During the resting state scan all subjects were instructed to stay as motionless as possible with their eyes closed and to not think of anything in particular. Additionally, for spatial normalization, each participant received a 3D anatomical MRI image with a T1-weighted magnetization-prepared rapid-acquisition gradient echo (MP-RAGE). The protocol was as follows: sagittal, repetition time = 2000 ms, echo time = 2.26 ms, inversion time = 900 ms, flip angle = 8°, slice thickness = 1 mm, FOV = 256 × 256 mm2, in-plane resolution = 1 × 1 mm2.

### Data preprocessing

Functional MRIs were preprocessed using SPM8 and in-house programs on the Matlab 2010 platform. Of the 180 scans obtained for each participant, the first 5 were discarded to allow the MR signal to reach a steady state. The remaining fMRI scans underwent preprocessing that included slice timing, motion correction, registration and normalization to a standard Montreal Neurological Institute (MNI) space, and resampling to a stereoscopic 3 mm3. The resulting data were checked to ensure alignment and spatial correspondence. Next, any linear trend shifts and head motion were removed voxel wise by regression. Finally, to reduce low-frequency drift and high-frequency noise, the fMRI data were temporally filtered using a band-pass range between 0.01 and 0.08 Hz with AFNI (http://afni.nimh.nih.gov/). It is noted that the spatial smoothing was omitted to maximize resolution in the analyses of thalamus nuclei groups, which are smaller brain structures. Additionally, we found that the average BOLD signal across all voxels in the brain was significantly correlated with the average time series within the thalamus as reported in other studies [22, 23]. These results confirm that the study did not regress out of the global signal within the brain.

### Cortical ROI definition

**Fig 1:**
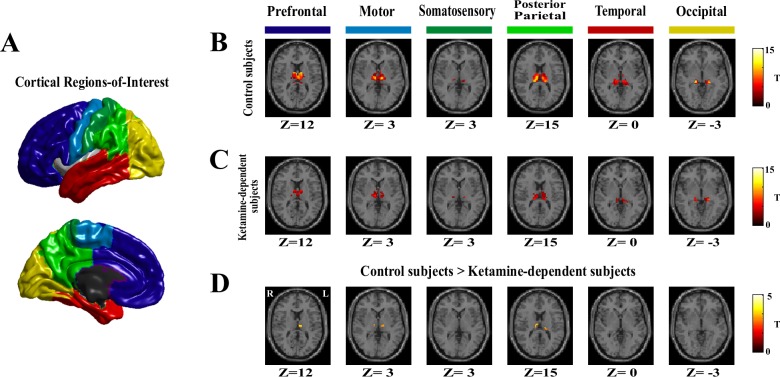
The diffusively decreased thalamocortical connectivity in ketamine-dependent subjects. A. The 6 cortical regions-of-interest. B. Thalamocortical connectivity of the drug-free control subjects. C. Thalamocortical connectivity of the ketamine-dependent subjects. D. Group differences in thalamocortical connectivity for the drug-free control subjects and the ketamine-dependent subjects. All shown clusters in this study were corrected for multiple comparisons with the corrected statistical threshold of P<0.05 using the AlphaSim program (single voxele p value<0.01, and ≥108 mm3 (4 adjacent voxels). A presentation formation referred to the previous study [26].

To investigate the specific functional relationships between the cortex and the thalamus, nuclei groups within the thalamus were partitioned into several nuclei groups using the methods proposed in previous studies [22, 24]. Since there are distinct connections between thalamic sub-regions and the cerebral cortex, specific functional relationships between them can be used to parcel the thalamus into nuclear subdivisions. In concurrence with relevant research knowledge [22, 24, 25] this study divided the cortex into 6 non-overlapping regions of interest: the prefrontal cortex, motor cortex/supplementary motor area, somatosensory cortex, temporal cortex, posterior parietal cortex, and occipital cortex. We used the Harvard-Oxford maximum probabilistic atlas of cortical structures (http://www.fmrib.ox.ac.uk/fsl/) to construct these regions of interest as shown in a previous study [26]. The cortical regions of interest (ROIs) are shown in **Fig 1A**, along with the details of the six regions of interest listed in S1 Table. In short, the prefrontal cortex ROI consisted of the superior, middle, and inferior frontal gyri, the middle and lateral orbitofrontal gyri, the gyrus rectus, and the anterior cingulate gyrus. Additionally, the motor cortex/supplementary motor area ROIs consisted of the precentral gyrus and the supplementary motor area. The somatosensory region of interest was the postcentral gyrus. The temporal lobe region of interest consisted of the superior, middle, and inferior temporal gyri, the parahippocampal gyrus, and the fusiform gyrus. The posterior parietal region of interest consisted of the superior parietal, the supramarginal, the angular gyri, the posterior cingulate, and the precuneus. Finally, the occipital region of interest consisted of the superior, middle, and inferior occipital gyri, the lingual gyrus, and the cuneus.

### Partial correlation mapping between cortical regions-of-interest and thalamus

The average fMRI time course was extracted from each cortical ROI. Partial correlations were computed for each voxel in the thalamus using these time courses. Specifically, after eliminating the influence of all other cortical ROIs, the results show a partial correlation between the local thalamic signal and the cortical ROI. To calculate statistical significance partial correlation coefficients were converted to a normal distribution using Fisher’s R-to-Z transformation. From this conversion we computed partial correlations between each thalamic voxel and each of the six cortical ROIs for each subject.

### Group-level Statistical analysis

For both ketamine-dependent subjects and drug free healthy controls we checked the thalamocortical connectivity patterns between the six regions of interest in the cortex compared to the distinct areas of the thalamus using a one-sample t-test. Then, we used a two-sample t-test to investigate whether there were significant differences in the thalamocortical connectivity patterns between the two groups. In this study, the AlphaSim program of the AFNI software package was used to correct for multiple comparisons. Parameters were as follows: single P value = 0.01, FWHM = 0, cluster connection radius r = 6.00 mm, mask of the thalamus with a resolution of 3*3*3 mm, and a number of Monte Carlo iterations = 1000. Finally, we explored whether there was a significant Pearson’s correlation between the strength of partial correlation and various ketamine use variables and behavioral scores in the ketamine dependent subjects. Behavioral scores included duration of ketamine abuse, daily average use of ketamine, total quantity of ketamine use, ketamine craving scores and cognitive test scores.

## Results

### Demographic characteristics

One ketamine dependent subject and one control subject had severe head motion and as a result were not included in statistical analysis. For other participants, the maximum displacement in the cardinal direction was not greater than 3 mm and the maximum spin was not greater than 3°. Therefore, the final data set consisted of one hundred and twenty-eight (40 ketamine dependent subjects and 88 drug-free control subjects) subjects. Detailed demographic and clinical characteristics for the two groups (40 ketamine dependent subjects and 88 drug-free healthy subjects) are summarized in **Table 1**.

**Table 1:** Demographic and drug use characteristics of ketamine dependent subjects and drug-free healthy subjects.

	Ketamine users/smokers (n = 40)	Controls (n = 88)	Two sample T test	X^2^
**Demographic variables**				
Age, years, mean±SD	26.8 ±4.91	27.1 ±5.14	p = 0.731	
Range (years)	19–39	19–39		
Male/Female, %	33/7 (17.5%)	70/18 (20.45%)		p = 0.696
Subjects’ education, years, mean±SD	11.9±2.75	14.1±2.94	P<0.001	
Right/left-handed, %	39/1 (2.56%)	85/3 (3.53%)		P>0.999
Unmarried/Married, %	25/15(37.5%)	54/34 (38.6%)		P = 0.902
**Ketamine use variables**				
Age of first use, years, mean±SD	23.10±5.21	―		
Range (years)	14–36	―		
Duration, months, mean±SD	41.1±21.79	―		
Range (months)	12–126	―		
Times of using ketamine/day	1.85	―		
Range (times)	1–4	―		
Quantity of using ketamine/time (g)	0.74 ±0.52	―		
Range (g)	0.1–2.5	―		
**Smoking variables**				
Smokers (>100 cigarette lifetime)	44	44		
Age of first smoking, years, mean±SD	15.2±3.09	17.9±4.29	P = 0.001	
Range (years)	10–30	11–30		
Duration, years, mean±SD	11.6±4.72	10.4±5.67	P = 0.286	
Range (years)	1.5–21	1.5–21		
Smoked cigarette/day,mean±SD	16.6±7.71	10.1±11.54	P<0.001	
Range (cigarettes)	8–40	10–40		
Other Drugs and alcohol use^a^				
Alcohol^b^	30	48		
Ecstasy	28	―		
Ma Gu (amphetaminecaffeine)	27	―		
Methamphetamine(ice)	23	―		
Marijuana	8	―		
Benzodiazepine (only diazepam)	6	―		
Heroin	―	―		
Cocaine	―	―		
**Drug craving**				
Ketamine craving (cm)	6.3±2.72	―		
Smoking craving (cm)	5.4±2.18	6.5±1.25	P = 0.016	
**Cognitive Tests**				
Digit Symbol Test (number of symbols)	64.5±15.83 (n = 40)	76.43±16.54 (n = 88)	P<0.001	
Trail Making Test A (seconds)	39.7±11.80 (n = 39)	39.3±12.57 (n = 88)	P = 0.883	
The Stroop Word Test (seconds)	66.7±15.78 (n = 37)	57.5±12.72 (n = 88)	P = 0.001	
The Stroop Color Test (seconds)	174.5±53.81 (n = 32)	138.5±39.74 (n = 85)	P<0.001	

^a^Each person could have tried more than one drug; drugs have been included even only tried once in the lifetime.

^b^Four participants reported drinking more than once/week among ketaminesubjects, and three control subjects reported drinking more than once/week.

### Thalamocortical connectivity

#### Drug-free Control subjects

Each cortical region of interest was connected to distinct, largely non-overlapping regions of the thalamus, as shown in **Fig 1B**. Although our methods in this study were not identical to previous studies the results are virtually identical [24, 26]. Specifically, the prefrontal cortex was functionally correlated to the anterior and dorsomedial regions of the thalamus. The motor cortex/supplementary motor area showed strong correlation with ventral lateral portions of the thalamus. The somatosensory regions of interest showed significant connection to ventral posterior-lateral portions of the thalamus and the posterior parietal cortex was robustly connected with the posterior nucleus and the pulvinar. Additionally, the temporal lobe and occipital cortex directly correlated with posterior medial and lateral areas of the thalamus, which appears consistent with the medial geniculate nucleus and the lateral geniculate nucleus, respectively.

#### Ketamine dependent subjects

A similar pattern of functional connectivity between the cortex and thalamus was demonstrated in the ketamine dependent subjects as shown in **Fig 1C**. However, there were qualitative differences between the ketamine dependent subjects and the drug free control subjects. Particularly, in the ketamine dependent subjects the connectivity between the motor cortex/supplementary motor area, the posterior parietal cortex and the specific nucleus of the thalamus appeared markedly less weak.

### Group differences in thalamocortical connectivity

Group differences in thalamocortical connectivity were summarized in **Table 2** and shown graphically in **Fig 1D**. The ketamine dependent subjects demonstrated less connectivity between the specific nucleus of the thalamus and the cortical regions, including the prefrontal cortex, the motor cortex /supplementary motor area, and the posterior parietal cortex. No increased thalamic connectivity was observed for the ketamine dependent subjects in comparison with the control subjects.

**Table 2:** Differences in thalamocortical connectivity between these two groups (ketamine dependent subjects and drug-free control subjects).

Seed Region of Interest, Contrast, and Brain regions	Montreal neurological Coordinates (x,y,z)	Peak t value	Cluster size (mm^3^)
**Prefrontal**			
Healthy controls > ketamine dependent subjects			
Left medial dorsal nucleus	-3, -18, 9	3.72	270
**Motor/supplementary motor area**			
Healthy controls > ketamine dependent subjects			
Left ventral posterior lateral nucleus	-12, -18, 6	3.41	459
Right ventral lateral nucleus	12, -15, 3	3.34	135
**Posterior parietal**			
Healthy controls > ketamine dependent subjects			
Right pulvinar	9, -27, 6	3.72	243
Left pulvinar	-18, -33, 6	2.76	108
Left pulvinar	-18, -27, 12	3.87	378
Left medial dorsal nucleus	-3, -9, 3	4.32	108
Right ventral lateral nucleus	12, -3, 6	3.20	162
Right lateral dorsal nucleus	12, -18, 15	3.53	405

The ketamine dependent subjects group demonstrated significantly less connectivity between the thalamus and the cortical regions-of-interest, including the prefrontal cortex, the motor cortex /supplementary motor area, and the posterior parietal cortex. No increased thalamic connectivity was observed for the ketamine dependent subjects in comparison with the control subjects.

### Correlations between thalamocortical connectivity and individual behavior scores in ketamine dependent subjects

**Fig 2:**
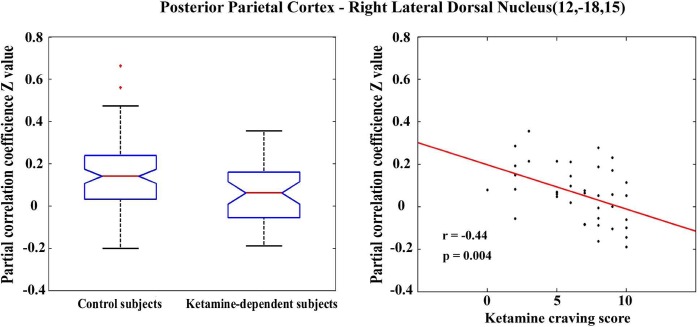
Functional connectivity patterns between the posterior parietal area and the right lateral dorsal nucleus (MNI (x,y,z) coordinate: (12, -18, 15)).

We also examined the relationship between connectivity changes and ketamine use related variables; these included age of first use (years), duration of use (months), frequency of use (how many times per day), quantity of ketamine use per time (grams), ketamine craving (cm) and scores of cognitive testing. We only found that the functional connectivity between the posterior parietal area and the right lateral dorsal nucleus (MNI [x, y, z] coordinate: (12, -18, 15)) was significantly correlated to individual ketamine craving scores (p<0.05, corrected) (Fig 2). We also calculated the correlation among all behavior scores and cognitive tests scores. We found that there are significant correlations between total ketamine intake and ketamine craving scores (Pearson’s r = 0.5963, p = 0.003), while there were no significant correlations between any behavioral and cognitive testing scores.

### Functional connection patterns of thalamic nuclei with the cortex

**Fig 3:**
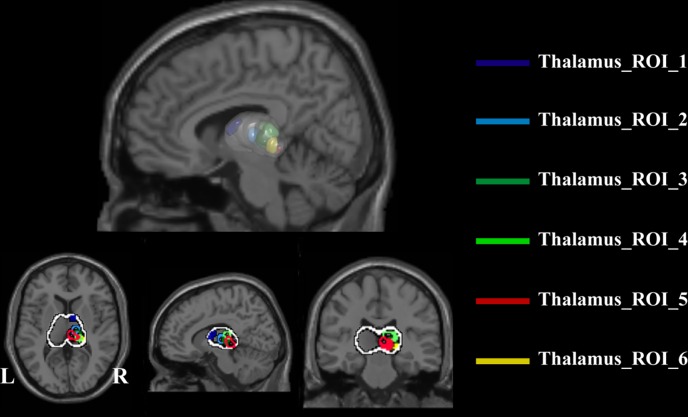
Location of thalamus subregion ROIs

**Fig 4:**
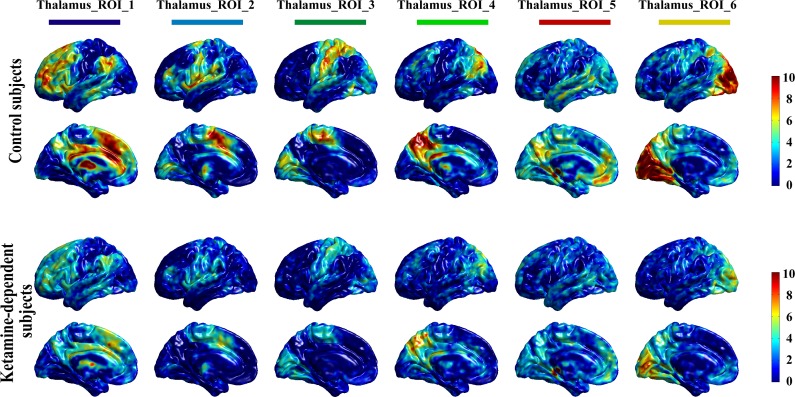
Functional connectivity patterns of thalamus ROIs.

**Fig 5:**
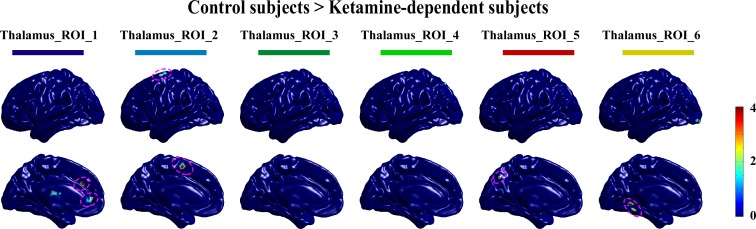
Differences of functional connectivity patterns of thalamus ROIs between drug-free control subject group and ketamine user group. The purple dotted lines show the significant different brain regions (single voxel P<0.01, cluster size>20 voxels).

We computed the functional connection patterns of thalamic nuclei with the cortex by first extracting the six thalamus subregions as ROIs, as shown in Fig 3. Specifically, in the T map of one sample t-test of the thalamocortical connectivity of the drug-free control subjects (as shown in Fig 1B in the main manuscript), the voxel with the peak T value and its surrounding region with a relatively large T value (75% ~ 85% of the peak T value for different ROIs) was extracted as an ROI. Only right thalami ROIs are extracted. Then, we checked that the six ROIs contained no overlapping regions. In detail, the ROI_1 (dark blue) was located at the anterior and dorsomedial regions of the right thalamus, the ROI_2 (light blue) was located at ventral lateral portions of the thalamus, the ROI_3 (dark green) was located at ventral posterior-lateral portions of the thalamus, the ROI_4 (light green) was located at the posterior nucleus and the pulvinar, and the ROI_5 (red) and ROI_6 (yellow) were located at posterior medial and lateral areas of the thalamus, respectively (Fig 3). Next, the average fMRI time course was extracted from each thalamus ROI. Using these time courses, partial correlations were computed for each voxel in the brain. Then, the one sample t-test was used to obtain connectivity patterns of each thalamus ROI, separately for the drug free control subject group and the ketamine user group, as shown in Fig 4. We think that these connectivity patterns are very consistent with our hypothesis, that is, different thalamic nuclei have significant distinctive connection patterns with the cortex. For example, the thalamus ROI_1 shows strong connections with the prefrontal cortex, while the thalamus ROI_2 shows significant connection with the primary motor cortex and the supplementary motor area. In summary, we believe these results of thalamocortical connectivity are relatively reliable and are grounds for further testing. Finally, we used a two-sample t-test to investigate whether there were significant differences in the functional connectivity patterns of thalamus ROIs between the two groups. The significant decrease in thalamocortical connectivity in the ketamine user group is represented by the dotted purple lines in Fig 5. These brain areas with significantly decreased thalamocortical connectivity in ketamine user group were almost located in the corresponding cortex ROIs.

## Discussion

This study, to the best of our knowledge, is the first to investigate resting state functional connectivity of the thalamus in chronic ketamine users compared to demographically matched drug free healthy subjects. Our findings provide first evidence that chronic ketamine users show abnormal thalamocortical connectivity of resting state brain activity when compared to healthy controls. We found that ketamine dependent subjects show significantly less connectivity between the thalamus and the cortical regions-of-interest, including the prefrontal cortex, the motor cortex /supplementary motor area, and the posterior parietal cortex. However, no increased thalamic connectivity was observed in the ketamine dependent subjects in comparison with the control subjects.

Although the current study is the first to demonstrate the changes of RSFC with chronic ketamine administration, previous studies with acute ketamine administration also showed changes of RSFC in healthy subjects. For example, using rsfMRI, a randomized, placebo-controlled, double-blind, crossover study demonstrated that acute ketamine administration markedly reduced RSFC of the default mode network (DMN) to the dorsal nexus (DN), the pregenual anterior cingulate (PACC) and medioprefrontal cortex (MPFC) via its representative hub, the posterior cingulate cortex (PCC) in healthy subjects compared to placebo [27, 28]. However, another similar RSFC study revealed that the administration of a subanesthetic dose of ketamine significantly increased cortico-thalamic connectivity of the somatosensory and temporal cortex [29]. One possible reason for this inconsistency may be due to the purpose of the study, the former is for treatment-resistant depression and the latter is for a schizophrenia model. Thus, these two studies focused on different circuit connectivity using similar methods.

Considering that addiction is a chronic, relapsing brain disease and a state of compulsive drug use, it can include tolerance, dependence and withdrawal symptoms [30]. And the disrupted circuit connectivity in addictive drug users may inform specific neurobiological substrates associated with addiction.[31]. Here, we discuss results from additional RSFC studies dealing with other types of substance addiction. A line of study demonstrated decreased functional connectivity in addiction of other drugs (such as cocaine and heroine). For example, a study on cocaine administration followed by fMRI observed decreased functional connectivity in the human primary visual and motor cortex after acute administration of cocaine [32]. In addition, Kelly et al. found reduced prefrontal interhemispheric resting-state functional connectivity (RSFC) in cocaine-dependent participants compared to control subjects [33]. By whole-brain resting-state fMRI connectivity analysis, Gu et al. reported almost universally reduced functional connectivity strength for six functional networks regions (ventral tegmental area, nucleus accumbens, the mediodorsal nucleus (MD) of the thalamus, amygdala, hippocampus and rostral anterior cingulate cortex) in cocaine users. They also found that the MD thalamus seed yielded decreased rsFC within extensive striatal regions and impaired mesocorticolimbic circuits for the cocaine group [34]. These regions are thought to be important for focusing and maintaining desired behaviors while suppressing unwanted behaviors [35]. Drug dependent subjects are known to have difficulties with response inhibition, which likely contributes to the propensity to relapse in the presence of drug related cues. However, both decreased and increased functional connectivity have been reported in heroin users. For example, Ma et al. found increased functional connectivity between the nucleus accumbens and ventral/rostral anterior cingulate cortex (ACC), the nucleus accumbens and orbital frontal cortex (OFC), the amygdala and OFC, and decreased functional connectivity between prefrontal cortex and OFC and between prefrontal cortex and ACC [36]. Additionally, evidence shows increased functional connectivity in the right hippocampus and decreased functional connectivity in the right dorsal anterior cingulate cortex and the left caudate in the default mode network (DMN) [37] in 14 chronic heroin users compared with 13 non-addicted controls. Using graph theory analysis (GTA), Liu et al. found dysfunctional brain connectivity among several brain regions in the network of chronic heroin users that may contribute to decreased self-control, impaired inhibitory function, as well deficits in stress regulation in twelve chronic heroin users when compared with twelve controls [38].

However, these are only a few studies that have examined addiction-related alterations in thalamocortical connectivity directly. Thalamocortical integration deficits in instrumental learning and performance could contribute to drug addiction as it often causes deficits in cognitive and emotional processes [14]. With 54 cocaine dependent patients and 54 age and gender matched healthy adult subjects, a simple reaction time task-fMRI study showed alteration of error-related thalamic- ventral medial prefrontal cortex connectivity, which is associated with impaired self-control in patients with cocaine dependence [39]. An animal study with electroencephalography (EEG) examined thalamocortical function in vivo and in vitro in mice after a cocaine “binge” administration. It illustrated that thalamocortical dysfunctions in a cocaine hydrochloride “binge” might be observed as a result of two distinct but additive events: 1. An increase in low frequency oscillatory thalamocortical activity and 2. Over-inhibition of ventrobasal neurons that can abnormally “lock” the whole thalamocortical system at low frequencies, ultimately inducing a thalamocortical dysrhythmia-like state [40]. Considering the key role of the thalamus in information transmission, processing, and basic corticocortical communication, examining the possible roles of the thalamus in addiction, as well as identifying open questions and exploring ways to address them may provide a more precise understanding of the pathophysiological mechanisms of addiction. Given the multifaceted nature of this complex brain disease, circuit disruptions, such as abnormal thalamocortical connectivity in the current study, may represent one of the potential targets for future treatment development.

In this study we also found that the functional connectivity between the posterior parietal area and the right lateral dorsal nucleus was significantly correlated to individual ketamine craving scores (p<0.05, corrected). Considering that craving is a central driving force for ongoing drug use and relapse following abstinence, using neuroimaging and other approaches to explore the neurobiology of craving is of utmost importance. Functional brain imaging in humans and animals has revealed an interconnected set of cortical and limbic brain regions that are involved in associative learning and prove to be an underlying theme for craving and relapse (see review [41]). However, we still do not fully understand the neurobiology and neurocircuitry mechanisms involved in drug-related craving.

Our study has several strengths, such as a relatively large sample size, use of the human chronic ketamine administration model, and the hypothesis-driven investigation of thalamocortical functional connectivity. However, we also acknowledge several limitations. First, although ketamine use participants did not meet dependence for any other substances except nicotine they may have engaged in other substance use and as such would be different from the controls in this regard. Second, the ketamine and control groups differed in smoking status and education. Although our analyses suggested that decreased thalamocortical functional connectivity observed are unlikely to reflect group differences in smoking and educational levels, future studies should match groups on smoking status and levels of education. Third, as is typical of studies in drug dependent populations, the sample was predominantly male. The potential role of gender differences should be examined in future studies. Additional limitations are demonstrated in the cognitive testing between the two groups, which future studies should take into consideration and instead match groups educationally in order to rule out any confounding factor.

In conclusion, this study provides first evidence for abnormal thalamocortical connectivity of resting state brain activity in chronic ketamine users. We found that the ketamine dependent subjects showed significantly less connectivity between the thalamus and the cortical regions-of-interest, including the prefrontal cortex, the motor cortex/supplementary motor area, and the posterior parietal cortex. However, no increased thalamic connectivity was observed for the ketamine dependent subjects in comparison with the control subjects. Further understanding of the pathophysiological mechanisms of the thalamus in addiction, specifically ketamine addiction, may facilitate the evaluation of much needed novel pharmacological agents for improved therapy of this complex disease.

## Supporting Information

S1 Table The brain areas within the 6 regions of interest. (DOCX)
