# Supplementary material for: Decreased Thalamocortical Connectivity in Chronic Ketamine Users
Source: PLoS One. 2016 Dec 15;11(12):e0167381. doi: 10.1371/journal.pone.0167381 (PMC5157971; doi:10.1371/journal.pone.0167381)
Supplement: S1 Table — (DOCX) [file pone.0167381.s001.docx]

S1 Table. The brain areas within the 6 regions of interest.

| **Region of Interest** | **Brain areas** |
| --- | --- |
| Prefrontal cortex | Frontal Pole  Superior Frontal Gyrus  Middle Frontal Gyrus  Inferior Frontal Gyrus, pars triangularis  Inferior Frontal Gyrus, pars opercularis  Frontal Medial Cortex  Subcallosal Cortex  Paracingulate Gyrus  Cingulate Gyrus, anterior division  Frontal Orbital Cortex  Frontal Operculum Cortex |
| Motor cortex/supplementary motor area | Precentral Gyrus  Juxtapositional Lobule Cortex  Central Opercular Cortex |
| Somatosensory cortex | Postcentral Gyrus |
| Temporal lobe | Temporal Pole  Superior Temporal Gyrus, anterior division  Superior Temporal Gyrus, posterior division  Middle Temporal Gyrus, anterior division  Middle Temporal Gyrus, posterior division  Middle Temporal Gyrus, temporooccipital part  Inferior Temporal Gyrus, anterior division  Inferior Temporal Gyrus, posterior division  Inferior Temporal Gyrus, temporooccipital part  Parahippocampal Gyrus, anterior division  Parahippocampal Gyrus, posterior division  Temporal Fusiform Cortex, anterior division  Temporal Fusiform Cortex, posterior division  Temporal Occipital Fusiform Cortex  Planum Polare  Heschl's Gyrus  Planum Temporale |
| Posterior parietal cortex | Superior Parietal Lobule  Supramarginal Gyrus, anterior division  Supramarginal Gyrus, posterior division  Angular Gyrus  Cingulate Gyrus, posterior division  Precuneous Cortex  Parietal Operculum Cortex |
| Occipital lobe | Lateral Occipital Cortex, superior division  Lateral Occipital Cortex, inferior division  Intracalcarine Cortex  Cuneal Cortex  Lingual Gyrus  Occipital Fusiform Gyrus  Supracalcarine Cortex  Occipital Pole |
